# Toward ethical provenance tracking: The GA4GH model data access agreement (DAA)

**DOI:** 10.1016/j.gim.2025.101594

**Published:** 2025-10-01

**Authors:** Alexander Bernier, Bartha Maria Knoppers, Jonathan Lawson, Robyn McDougall, Maili Raven-Adams, Vasiliki Rahimzadeh

**Affiliations:** 1 University of Toronto, Faculty of Law, Falconer Hall, 84 Queens Park, Toronto, ON Canada; 2 McGill University, Faculty of Medicine and Health Sciences, Department of Human Genetics, Montreal, Quebec, QC, Canada; 3 Broad Institute of Harvard and the Massachusetts Institute of Technology, Cambridge, MA; 4 Gilbert’s LLP. Toronto, ON, Canada; 5 Wellcome Sanger Institute, Wellcome Genome Campus, Hinxton, Cambridge, United Kingdom; 6 Baylor College of Medicine, Houston, TX

**Keywords:** Comparative law, Data access agreement, Data access oversight, Data stewardship, Empirical bioethics

## Abstract

**Purpose:**

Standardizing contractual clauses that govern data access enables research institutions to responsibly steward genomic and related health data while enabling its efficient downstream reuse.

**Methods:**

We describe a document analysis study using both qualitative and comparative law analytical approaches to identify the most common categories of clauses from 29 different data access agreements used by human biomedical research consortia globally. We furthermore characterized the legal positions and standard practices for each common element of the agreement and synthesized across them to develop model clauses. A total of 3 discussion sessions were organized virtually to refine the clauses among members of the Ethical Provenance Subgroup of the Global Alliance for Genomics and Health.

**Results:**

We developed 15 unique data access clauses corresponding to the most common legal elements identified in the sampled agreements.

**Conclusion:**

Model clauses can be used to drive administrative efficiencies and institutional compliance for managing access to human genomic data for research. Additional machine-readable consents and software solutions are needed to support traceable “ethical provenance” of human genomic data and communicate data use conditions throughout the data’s life-cycle.

## Introduction

Scholars have previously reported that negotiating the contractual terms of data access agreements (DAAs) remains a rate-limiting step for the release of controlled-access research data.^1^ DAAs outline the specific terms by which data may be used and shared across institutions and users. Data stewards use DAAs to guarantee that third parties respect the ethical, legal, and organizational restrictions on data use. Because DAAs are often developed bespoke on a consortium-specific or repository-specific basis, negotiating them can be administratively burden-some. Moreover, underresourced institutions may lack the ability to develop DAAs specific to their institution, hindering their ability to more widely share data with external researchers, and to access data sets they may require from other institutions. Standard DAA clauses would help to address these administrative burdens and support more consistent data access and management practices across research institutions. Such standards must align with best practices in data governance and ethics, while also remaining responsive to population-specific governance needs and to local variations in laws, policies, and practices.

The Global Alliance for Genomics and Health (GA4GH) created the Ethical Provenance Subgroup (EPS) in 2022 to address these recurring data governance challenges and innovate practical solutions that advance the human right to benefit from science. Ethical provenance generally refers to a “combination of technological and organizational structures that enable groups of researchers to compare and monitor the national biomedical ethics requirements, legal commitments and institutional conditions of use applicable to their respective data sets, despite these rules being expressed in the incommensurable language of distinct normative frameworks.”^2,3^ To this end, the EPS aims to help standardize practices, tools, and processes—including but not limited to DAAs—used to trace data use conditions throughout the research life-cycle, from data generation, through contribution to a steward, to reuse.

In this article, we first explain the relevance of ethical provenance to data access oversight and describe the empirical process we used to develop model DAA clauses for human genomic and related data sharing. We then report the outputs of a document analysis that identifies common elements of DAAs and characterize the legal positions taken by international biomedical research consortia on significant issues of public policy that affect how data can be reused. This analysis assessed the degree to which existing contractual models are aligned. We propose 15 model contractual clauses that we believe can help research institutions share data according to compatible conditions of future use (Supplemental Information 1). We conclude with recommendations for how distinct actors in the biomedical research environment, such as researchers, funding agencies, and data repositories, could foster the transition toward standardized and machine-readable contract language and highlight remaining questions that motivate future research in this space.

### Ethical provenance and data access oversight

Research institutions, data repositories, and research consortia could enhance respect for contributor-defined data use conditions and reduce administrative burdens if (1) elements of institutional data access and sharing practices were standardized and (2) data use conditions were recorded and tracked using compatible processes across institutions.^4^ Efforts to systematize the representation of data use conditions in service of ethical provenance are underway. The use of consensus language to describe data use conditions facilitates compliance. Similarly, ensuring data use conditions are consistently interpreted across multiple institutions, from data generation to data destruction. We refer to the recording, tracking, and enforcement of contributor-defined terms of data use according to consistent conventions as “ethical provenance.”^2,3^

Recording and tracking ethical provenance creates several benefits. It provides a chain of evidence that data use respects the applicable conditions communicated at the time of consent, as well as data sharing policies and DAAs. Repositories and research institutions can use ethical provenance records to describe conditions of use through natural language or ontology terms, which are preserved with the data as appended policies and metadata terms. Breach of data use conditions can be sanctioned using both formal and informal mechanisms. These can include reputational sanctions, the denial of continued research cooperation, and contract enforcement. Incentivizing compliance using formal or informal mechanisms, however, is predicated on the consistent interpretation of applicable data use conditions as data are transferred and reused. Ethical provenance responds to challenges in representing and communicating such use conditions in a consistent manner, using cost-effective mechanisms, and in reducing compliance, monitoring, and enforcement costs.

In tracking ethical provenance using shared conventions, data stewards can consistently interpret data use conditions across distinct jurisdictions. In so doing, data stewards secure credible commitments from data users to respect the applicable data use conditions without the need for centralized oversight and monitoring.^5^ This is important because no central steward bears full responsibilities for the definition and enforcement of all data use conditions. Global commitment to tracking ethical provenance according to compatible practices is required to transition from the top-down, centralized administration of scientific activities, toward decentralized and federated open science research ecosystems. Without cost-effective mechanisms to communicate and represent data use conditions, independent actors cannot coordinate the monitoring and enforcement thereof. The centralized development, communication, and enforcement of such use conditions would thus be required because the administrative costs of coordinating on decentralized enforcement would be prohibitive. Tools that facilitate the decentralized tracking of ethical provenance are needed to minimize reliance on central monitoring and enforcement bodies for this reason.

In summary, data contributors, data stewards, and the end users of research data need common ways to represent and communicate conditions of future data use in a consistent and transaction-cost-efficient manner, which support decentralized data exchange. To make progress toward this vision, we developed model clauses which could reduce the administrative costs of exchanging data across multiple institutions, if subject to widespread adoption. We chose to work toward the alignment of contract language in particular because difficulties in negotiating and interpreting contracts is one of the most often-cited barriers to seamless data exchange.^1^

We note that the use of the word “institutions” in this manuscript follows the conventions of the bioethics literature—referring to organizations and networked subsets thereof working toward common purposes—rather than the conventions in the institutional economics literature. For an institutional economics readership, the term “institutions” as used here is a closer approximation to the term “organizations” in the institutional economics literature.

### Materials and Methods

We issued an open call for DAAs to all members of GA4GH in April 2023. We also purposively selected (inter) national data repositories which, according to our prior knowledge, managed access to genomic and related health data. To support global representation in our data set, we targeted outreach to known representatives of genomic data repositories participating in GA4GH, within geographic regions traditionally underrepresented in genomic research. We supplemented agreements collected via this broad call with publicly available DAAs downloaded online from institutional webpages of GA4GH Driver Projects, the National Institutes of Health (NIH), and other consortia and data repositories that met our inclusion criteria.

All DAAs that met at least 1 among the following inclusion criteria were reviewed by 2 analysts (A.B., R.M.):

- Agreements dedicated to the sharing of data from single studies, from consortia comprised from multiple interrelated studies, and from repositories hosting data from multiple independent studies and consortia.
- Agreements from GA4GH Driver Projects and their host organizations.
- Agreements from NIH-funded projects.
- Agreements related to large-scale and small-scale studies, consortia, and repositories.
- Agreements related to genomics, and of other important research areas, such as population health, neuroscience, and epidemiology.
- Agreements related to distinct populations (eg, indigenous populations) and global representation.

The agreements evaluated all authorized 1 research institution to access and download data supplied by another research institution (ie, these were all traditional DAAs). Non-DAA contractual agreements, such as data contribution agreements or multilateral data sharing agreements, were excluded from the analysis. The excluded categories of agreements enable data to be deposited in a repository, and enable reciprocal data exchange between multiple institutions, respectively. Agreements pertaining to model-to-data analysis, or distributed computation, were likewise excluded. Non-DAA agreements were excluded because their language addresses different public policy considerations than are outlined in the terms of DAAs. Including such contracts would have confounded the main analysis, making it difficult to draw firm conclusions about the common features of DAAs. It would have been impossible to determine which differences reflected differing public policy priorities and choice of legal instruments in addressing a selfsame contractual issue, and which reflected more foundational differences in the function of the contract.

### Data analysis

Four of us with legal training (A.B., B.M.K., M.R.A., R.M.) collected 29 total DAAs and reviewed each agreement for common elements using content analysis.^6,7^ We then created a list of common contractual elements, which was further refined in collaboration with EPS members. Clauses considered important, despite being uncommon in the DAAs reviewed, were added to the preliminary list. Distinct clauses with subject-matter overlap were collapsed into 1 category. Two of us (A.B. and B.M.K.) then collaboratively reviewed each DAA and classified clauses into their relevant thematic categories using Microsoft Excel and Dedoose. Prior studies geared to developing standard-form data sharing agreement clauses for specific populations or toward performing comparative evaluations were also considered in developing our own evaluative criteria, and in selecting the categories of clauses to be evaluated.^8,9^ In addition to categorizing each contractual clause according to the major issues it addressed, we created labels indicating the specific legal approach taken to address the contractual issue. These secondary labels allowed us to capture major differences in how distinct contract design choices affected genomic data access and use. The resulting classifications and limited metadata about the agreements are detailed in full in Supplemental Information 2.

We considered the following desiderata when drafting model clauses:

- Prevalence of the approach among the examples compared (ie, evidence of existing adoption).
- Clarity and concision.
- Potential to foster efficiencies and reduce costs of contract administration, monitoring, and enforcement.
- Respect for key international bioethics principles.
- Congruence with open science principles and scientific attribution mechanisms.
- Preservation of confidentiality and security of sensitive human biological data.
- Prevention of harm and stigmatization directed toward vulnerable populations and communities.
- Translatable across distinct legal traditions.
- Compatibility with existing GA4GH and NIH policies and best practices.

The draft model clauses are compatible with existing best practices, and reflect the desiderata identified above, in a nonhierarchical and holistic manner. We circulated a complete list of the draft model clauses to all members of the EPS. To systematize member feedback and generate substantive discussion, we held 3 virtual working group meetings from January to April 2024. Each draft clause was discussed in turn and revised based on member feedback. After internal EPS revisions, we invited GA4GH-wide comments on the clauses during a month-long public consultation period. Table 1 summarizes the changes made to the original draft clauses based on feedback from EPS members.

## Results

We synthesized 15 model DAA clauses in cooperation with the EP workgroup from *N* = 29 data access agreements (see Supplemental Information 1 for full list of clauses, and Table 2 for aggregate data on geographic provenance, population, and disease group). In the sections that follow, we first explain the legal “function” of each draft clause and describe notable findings drawn from our empirical analysis of sampled clauses. These descriptions summarize the competing approaches observed across the agreements studied. Second, we provide justifications for the language used in the model clauses. These are not intended to provide a normative justification for the choices made. Rather, they explain the central public policy considerations which motivated our choice of model clause language. They describe how the output clauses integrate the desiderata above and how we decided to balance competing priorities in choosing among available alternative clauses.

### Purpose of use

#### Clause description and notable empirical findings

“Purpose of Use” clauses describe how data can be used and the general commitments to respect in using it. This clause takes the data use conditions applicable to data and turns them into binding contractual obligations. Almost all agreements limited permissible data use to the research purposes described in the agreement or its supporting documents. Some individual agreements incorporated additional context-specific restrictions on use, such as limiting use to specific disease categories or research questions, or explicitly precluding the data’s commercial use, its clinical use, or its use in criminal proceedings.

#### Justification for model clause

A “Purpose of Use” clause is not difficult to draft on a case-specific basis for a specific contractual agreement. It is, however, challenging to turn into a generalizable template clause, because its substantive contents respond to context-specific governance considerations. Our Purpose of Use clause refers to an Annex in which the data steward(s) can detail the applicable data use conditions using their own policies, plain text, or ontology terms. Using common contractual templates drives economies of standardization for data stewards. It reduces the costs of drafting, negotiating, interpreting, and enforcing contracts. Nonetheless, granting data stewards the freedom to associate bespoke data use conditions to their data is important, because these often change to reflect population-specific, jurisdiction-specific, or organization-specific requirements. In other words, ours is an effort to streamline the mechanisms used to communicate and enforce the conditions of data use applied to research data. We are not attempting to dictate the specific purposes for which data use should be authorized. This latter choice rests with research participants, researchers, and data stewards.

### Reporting and monitoring of use and access

#### Clause description and notable empirical findings

Data stewards enact 2 axes of control when managing controlled-access data release. First, they impose access conditions that limit data access to authorized parties with appropriate credentials that intend to engage in permitted data use(s). Second, they impose ongoing monitoring, audit, and reporting requirements that hold authorized users accountable to the commitments made upon requesting access and signing access agreements. “Reporting and Monitoring of Use and Access” clauses communicate these latter, ongoing, responsibilities to research organizations, principal investigators (PIs), and their research teams. The clauses that we reviewed varied with regard to the specific mechanisms used to perform these functions. Common mechanisms included requiring user PIs to supervise their research team’s use of the data, requiring research team members to read and to acknowledge the access agreement, and requiring data users to complete periodic or final reports detailing how the data were used.

#### Justification for model clause

Our model clause first requires that PIs understand the obligations in the agreement and make these transparent to all members of the research team who are granted access to the data. Second, it clarifies that legal consequences for non-compliance are restricted to the research organization that requests access to the data. PIs are required to acknowledge the terms of the agreement but cannot be sanctioned for their failure to respect it. Their incentives to respect these commitments instead arise from the reputational, professional, and intercollegial consequences of failing to uphold them. This approach balances the incentive-generating function of organizational legal liability, against the chilling effects that personal legal liability would have on researchers.

Third, its reporting sections require the notification of changes in the research team’s composition to the data steward and the submission of a final report at the end of the data access period. This ensures that data stewards are aware of who uses the information and of the scientific outcomes of its use, without encumbering both the steward and the authorized user with disproportionate administrative duties.

### Intellectual property requirements

#### Clause description and notable empirical findings

“Intellectual Property Requirements” clauses communicate (1) whether and how intellectual property rights are licensed or assigned to the data recipient, (2) the respective entitlement of each of the parties to claim intellectual property (IP) rights in downstream discoveries, and (3) other obligations that affect how parties can or must claim or exercise IP rights relative to the shared data or relative to downstream discoveries derived therefrom. Although no one singular approach was consistently adopted across all of the agreements studied, almost all agreements that addressed IP adopted a combination of the following elements: (1) asserting data steward ownership of upstream IP rights, (2) communicating to users that the data could be subject to third-party IP rights, (3) prohibiting users’ assertion of rights that could frustrate the use of the shared data, and (4) confirming to users that no rights in the data were transferred to them. Most agreements also made explicit that users could claim IP rights in their downstream discoveries occasioned through the use of the data.

#### Justification for model clause

Our “Intellectual Property Requirements” clause incorporates core open science tenets. It balances the right of the original data steward to keep shared data sets free from third parties’ IP rights, against the countervailing rights of third parties to assert IP rights on their own future data discoveries. Furthermore, it cautions the data user that the data steward or other third parties could hold IP or related rights in the shared data sets and that those rights are not transferred to the data user. IP rules vary across jurisdictions. Shared global approaches to bounding the rights and the interests of data contributors and data users have nonetheless emerged. This is made possible because open science standards world-round incentivize the release of existing data sets, while also encouraging third parties to commercialize their downstream discoveries.^10,11^ The incentive structure of our resulting “Intellectual Property Requirements” clause shields data stewards from legal liability, safeguards shared data sets from predatory uses of IP to encumber such public goods with private rights, and liberates end users to IP-protect and to commercialize their own discoveries.

### Outbound data transfers

#### Clause description and notable empirical findings

Clauses on outbound data transfers are used to ensure that controlled-access data are not shared with unauthorized third parties. Such clauses must prevent illicit data transfers that make the data steward liable, without precluding disclosures that the data user must make to ensure legal compliance or to complete ongoing research. Each contract we reviewed addressed this issue using a substantively identical approach, imposing a default preclusion on data transfers to third parties. A minority of the agreements reviewed also incorporated context-specific exceptions to this preclusion; eg, to enable compliance with audit or monitoring requirements or with applicable law. Our clause defaults to the preexisting consensus position in the agreements reviewed, while also incorporating those exceptions that are of general applicability.

#### Justification for model clause

The model “Outbound Data Transfer” clause sets a general rule against data transfers to other legal entities, or to researchers not named on the research team. Because these parties are not subject to the contract, the data steward would have no recourse against them for data misuse if data were disclosed to them. However, transfers to ensure compliance with audit or monitoring requirements (eg, those of clinical trial regulators or funding agencies) and public law are permitted. Transfers to third-party service providers are also authorized (eg, contracted providers of compute services or cloud storage). Precluding these could cause the agreement and the other disclosure requirements applicable to data users to conflict.

### Contract breach notification

#### Clause description and notable empirical findings

Contract breach clauses detail what measures must be taken to respond to a breach of contract. These clauses dictate both the follow-up measures that must be taken, and the respective rights and obligations of the parties in response. The DAAs we analyzed differed as to whether a contract breach creates a unilateral right to terminate the agreement. Some DAAs enable the data steward to terminate the agreement without requiring justification.

#### Justification for model clause

The model “Contract Breach Notification” clause requires the data user to inform the data steward if the agreement is breached. The data steward can terminate the agreement in cases which the breach is not remediated in reasonable time or in which the breach is irremediable. We avoided creating a strong, unilateral right to terminate because DAAs are relational contracts. The long-term, ongoing interactions between the steward and the researchers across data access events create strong incentives to respect the agreement.^12–15^ Punitive rights of termination are thus not necessary to motivate responsible data use. The promise of a continued relationship with the steward, and the professional norms of good scientific practice, provide sufficient incentives. Further, the researchers invest significant resources in analyzing data after accessing them. This investment has a property called “asset specificity”—the effort made in analyzing those data, or in improving it, cannot be repurposed elsewhere if the data are lost.^16^ For these reasons, giving the steward a right to end the contract unilaterally for small breaches would be disproportionate to the harm, if any, resulting from the breach. The researchers could lose months or years of effort without warning. This could prompt researchers not to request data at all. Risk of unilateral termination might also have a counterproductive effect in that researchers could be incentivized to conceal, rather than disclose, breaches.

### Confidentiality

**Clause description and notable empirical findings** Confidentiality clauses impose duties to hold information confidential on each individual and organization that accesses data. These reiterate and pass on the fiduciary responsibilities that researchers, medical professionals, and health care organizations owe research participants and patients.^17–19^ Each contract we reviewed addressed this issue using substantively identical language. Our clause defaults to the pre-existing consensus position in the agreements reviewed.

#### Justification for model clause

Our proposed “Confidentiality” clause precludes researchers from breaching the confidentiality of research participants, using the standard contractual language that has already achieved consensus approval.

### Reidentification and harm

#### Clause description and notable empirical findings

Confidentiality is an obligation that arises from the long-standing ethical duties of researchers and medical professionals toward their charges.^20,21^ Emerging scholarly perspectives on data protection, data privacy, and on broader societal harms arising from the misuse of aggregated data have led to the recognition of new, related ethical duties to avoid causing informational harms.^22,23^

These new duties include avoiding the reidentification of research participants and avoiding the use of data in a manner that could harm communities or groups. The existence of these ethical duties is well established and un-controversial. However, what remains unsettled is the strength of the contractual obligation that substantiates these duties. Is it enough to preclude direct attempts to perform individual reidentification or induce group harm? Should a consequentialist preclusion on acts that induce reidentification or group harm be preferred, absent an intent requirement? Is it safer still to outright preclude activities such as data combination or data linkage, which could induce reidentification or harm groups? The contracts we evaluated were sharply divided on this issue.

Most of the contracts reviewed precluded acts resulting in reidentification. Some precluded acts in general that could cause reidentification. Others precluded specific categories of acts in which these could cause reidentification. Still others precluded specific categories of acts, regardless of whether such acts posed a prospect of causing reidentification. A small subset of the clauses reviewed also addressed select emergent risks, such as site-level reidentification, harm and stigmatization of communities, or the identification of families, groups, or communities.

#### Justification for model clause

Our proposed model clause precludes acts that attempt to, or that should reasonably be anticipated to, cause reidentification, harm, or stigmatization. We include no outright bar on specified categories of research activities. This approach protects research participants without imposing disproportionate administrative burdens on researchers. This choice of language reflects and reinforces emergent academic consensus on avoiding the stigmatization of vulnerable communities that have been subject to historical exploitation by researchers and research institutions.^24^ It is not intended to preclude research that could prove controversial but rather to preclude prejudicial conduct thinly veiled as science.

### Scientific publication

#### Clause description and notable empirical findings

“Scientific Publication” clauses generally state what researchers must do in publishing their findings. The DAAs we evaluated incentivized data users differently from one another. For example, some required data users to share their own research outputs in open repositories, requiring open IP licenses to be applied to those outputs, or requiring researchers to offer coauthorship to the original research team that generated the data. Clauses regarding scientific publication were the least harmonized in our sample of DAAs. The requirement to preserve participant confidentiality was observed in the majority of the clauses reviewed. Other elements observed in the clauses were each represented in only a small subset thereof. Examples include obligations to respect the publication policies of the data steward, obligations to consult with the data steward before publishing findings, embargo periods restricting data users from publishing on the data for specified time, obligations to offer the data steward coauthorship, obligations to publish research findings, obligations to publish research findings openly, and obligations to avoid publishing in a manner that could breach individual or community privacy rights and rights to nondiscrimination or nonstigmatization. These heterogeneous results suggest that the topic of scientific publication would benefit from future consensus-building efforts.

#### Justification for model clause

Our model “Scientific Publication” clause requires that attribution statements should be associated to resulting research outputs in cases in which the Data Provider requests so. Second, it requires that the publication outputs do not create a significant risk of inducing individual reidentification or group harm.

### Data destruction

#### Clause description and notable empirical findings

“Data Destruction” clauses typically tell the data user when they must destroy the data. Although some commonalities were identified, the data destruction clauses we assessed differed in 3 important respects: (1) the conditions under which the data must be destroyed, (2) the exceptions to data destruction requirements, and (3) the alternative methods of data disposition available to the user, other than data destruction.

#### Justification for model clause

Our “Data Destruction” clause requires destruction upon the contract’s termination or the conclusion of research activities, whichever comes first. Data can be retained for compliance purposes or to ensure the integrity of ongoing studies. Instead of proceeding through data destruction, the data users can choose to archive or to anonymize data. However, proceeding through an alternate method of disposition requires approval from the data steward.

### Data security

#### Clause description and notable empirical findings

Data stewards must ensure that data users hold data secure. The DAAs we reviewed structure this obligation in one of 2 ways. Some agreements require that specified security measures be implemented. Others require data users to implement appropriate security measures but leave them to determine which specific measures to implement.

In drafting a clause on data security, security audits must also be addressed. That is, what data security audits the data steward can compel. The audit powers in the contracts reviewed range from the light-touch audit of security documentation to the more intensive audit of machines or of physical premises. Data stewards often bear the costs of audit.

#### Justification for model clause

Data users are better placed than data stewards to appreciate and mitigate the risks that their activities create. Therefore, the data user should choose which security measures to apply. Clauses that outline required security measures in exact terms are also more expensive to draft and to update than more general ones.^25,26^ Our “Data Security” clause creates a general obligation to implement appropriate security measures, without stipulating their contents.

Our “Data Security” clause further limits audits to those of security documentation. This reflects the disproportionate costs that coordinating more intensive audits could impose on both data stewards and data users. Light-touch audits can be more feasible (and therefore readily performed) for smaller research organizations. This helps ensure fairness of outcomes, even between research organizations with unequal resources and bargaining power.

### Data breach notification

#### Clause description and notable empirical findings

DAAs must further respond to unauthorized disclosures of data. A “Data Breach Notification” clause defines the powers and obligations of parties when an unauthorized disclosure occurs. Often, such a clause further clarifies what constitutes an unauthorized disclosure of data.

The DAAs we reviewed varied significantly in this regard. The definitions and consequences of a data breach differed widely. Some data breach clauses terminate the contract immediately upon breach. Others empower the data steward to end the agreement at its discretion if a breach takes place.

Data breach notification clauses also varied as to the “conservatory measures” that the data user must implement upon discovering a breach. Some required the data user to implement all measures that the data steward specifies after the occurrence of the breach. Others state the exact measures that are to be taken in the contract.

#### Justification for model clause

Our clause defines breach in the broadest possible manner. It captures known and suspected disclosures to unauthorized recipients, security lapses, and misuses on the part of authorized users. The model clause does not let the data steward unilaterally terminate the agreement after a data breach. The steward and the user must collaborate together to mitigate the data breach. However, the data user may only resume using the data after the breach is managed. Our clause also accepts that the data steward can notify affected persons, and regulatory authorities. This structure promotes cooperation between data users and data stewards in mitigating breaches and helps to ensure the continuity of affected research efforts.

### Changes to the agreement

#### Clause description and notable empirical findings

Those who write contracts cannot predict the future. Situations change, and formal contracts must often be updated to better match protean real-world circumstances. But too much flexibility takes the teeth out of an agreement. Parties, after all, use contracts to restrict each other’s future freedom to mutual benefit. Thus, DAAs often include terms for when, how, and why an agreement is subject to change.

The DAAs studied adopted distinct approaches to contract modification. Some authorize either of the parties to request a modification, leading to either acceptance or to contract termination. Others require changes to be made through mutual consensus. Still others create a unilateral right for the data steward to require contract modification, absent which it can terminate the agreement.

#### Justification for model clause

Our model clause authorizes the data steward to unilaterally modify the terms of the contractual agreement, requiring the data user to either accept the changes or terminate the agreement. This position appears on first reading to favor the interests of the data steward. However, there is a strong ethical motivation for this. The DAA is not a true bilateral contract negotiated between 2 parties, a data steward and data user, with equal bargaining power. Instead, the data steward acts as a proxy for the research participants from whom the data were derived.^27^ Research participants have limited say in data sharing arrangements after they consent and have little bargaining power. The data steward zealously protects their interests and acts on their data sharing choices. It is therefore instrumental that the DAA enable unilateral modification where needed to better protect research participants. Such changes might be required if laws are modified, if new risks to research participants materialize, or as data stewardship best practices evolve in light of changing norms.

The contract modification clause developed therefore enables the data steward to change the agreement as is required, to respond to changing governance needs. The data user can accept the modifications, or else accept the termination of the contractual agreement.

### Liability

#### Clause description and notable empirical findings

Drafting clauses that apportion liability between parties can be a pain point in the finalization of contracts. Each party wants to limit their own liability as much as possible. This is especially the case for acts that another party to a contract performs and that lie outside their control.

We observed that DAAs shielded the data steward from all liability. Data stewards further disclaimed guarantees of rights and permissions in the data. What might explain this bias in favor of the interests of data stewards? Data stewards drafted most of the agreements evaluated. Such stewards might seek to protect their own interests above end users. In the alternative, the bias in favor of data stewards could reflect efforts to limit their exposure to new risks that data users create. Because the data steward is a passive holder of data, and the end-user actively uses these data, the acts of the data user are more likely to cause the data steward to be liable than the inverse.

#### Justification for model clause

Our resulting “Liability” clause exposes both the data steward and the end data user to the damages that each generates. It does not provide either a full exculpation from liability. Nonetheless, because data stewards receive data from data contributors, the clause stipulating that the data are provided without warranties or guarantees was retained.

Laws, such as those of the United States, restrict research organizations from contracting into significant liability. Our clause thus limits the liability of parties to the maximum that local law permits, so that all parties from all countries can sign it.^28^

### Duration

#### Clause description and notable empirical findings

Most contracts have a duration clause that stipulates when the contract ends. Unexpectedly, a significant number of the DAAs evaluated did not include one. This could be because the data access approvals that the data steward or its data access committee (DAC) offer themselves bound the duration of authorized data access. These issues could also be addressed in access policies, descriptions of data use conditions, or other external documents.

#### Justification for model clause

Our model clause sets a default contract duration of 1 year. We chose 1 year because data access approvals are most often issued for this period of time, subject to renewal. Mirroring the duration of a data access approval in companion access agreements creates administrative efficiencies. Otherwise, the data access approval and the DAA would need to be renewed at different times. The default period of a data access authorization can differ across distinct access committees. Therefore, notes are left to the drafters of the agreement, encouraging them to update its duration to their specific circumstances.

## Discussion

We observed significant variation across the contractual clauses in the DAAs analyzed in this study. Each clause reflected a distinct balance of public policy commitments. Some clauses, for example, sought to protect the interests of data producers by imposing publication embargos, for example, or requiring that data producers be named as co-authors on peer reviewed papers. Other interests captured in DAA clauses advanced cultures of open science by liberating data sets from IP protections or requiring that research outputs be released fully open access.

### Balancing stakeholder interests

Clauses also balanced conflicting interests of data stewards, downstream data users, and research participants. Adjusting the legal obligations that these different parties bear changes their respective costs and modifies their incentives. Contracts structure the interactions of their users for the long-term. Third parties also feel its effects because contract terms shape the behavior of the co-contractants toward these external parties.

### Cost

Common template agreements are required to reduce the transaction costs of coordinating data exchanges across large networks of biomedical researchers. If parties draft bespoke contracts for each exchange, the costs of working together increase. The costs of interpreting multiple contracts and ensuring compliance with each also increase. Even for well-constructed agreements, the costs of interpretation and compliance balloon as parties are bound to more agreements. This provides strong justification to standardize DAA interpretation and execution processes in large-scale infrastructure science.

### Recommendations for practice

We propose several practical recommendations for how to encourage organizational adoption and effective use of the model DAA clauses developed. First, research organizations that share data should adopt compatible DAA clauses. The GA4GH Model DAA clauses we developed will be useful in this respect, but specialized contractual clauses to share data within specific jurisdictions, pathologies, or populations might still be needed. Second, research funding agencies and large, central, data repositories should adopt policies that nudge independent research organizations toward compatible contract language for data access. Funders could, for example, supply model contractual language, or mandate use of their own contract templates. Moreover, central data repositories could develop software tools that help formulate contracts and automate compliance. Third, research organizations could integrate contract signatures into procedural workflows used to review data access requests. The “library card” model^29^ is likewise efficient and allows applicant organizations to sign a DAA once, capturing all of the data access requests that researchers from that organization will make in the future rather than negotiating a bespoke contract for each data access request. Moving forward, the principal onus lies on organizations such as large-scale research networks, funders, and repositories to further instigate the shift toward interoperable, machine-readable contract language. These actors are best placed to incentivize this change and to measure progress toward realizing the benefits of ethical provenance for all current and prospective data contributors.

## Conclusion

Defining a clear, auditable chain of rights and permissions for data use from the moment of data generation to secure storage and subsequent sharing (ie, ethical provenance) is foundational to responsible data stewardship.^30^ Failure to harmonize or, at a minimum, ensure interoperability of data use permissions undermines participant trust. Participants entrust their data to researchers. By contributing their data, participants also have expectations that it will be used ethically. Once such trust is compromised, it cannot be easily regained. Our comparative analysis of DAAs from global biomedical research consortia within and external to the GA4GH suggests that common contractual elements can be standardized to support provenance tracking across organizations and borders. Furthermore, many agreements we analyzed balanced similar interests, including participant protection, benefit maximization, confidentiality, open science, and administrative oversight.

Additional empirical research is needed to assess the relative costs that alternate clause designs impose on data stewards, data users, and research participants. Further qualitative data about stakeholder preferences should also be collected. This information could be useful to update the template clauses in the future, to account for evolving stakeholder preferences, public policy reforms, and further technological innovations.

Interoperable contract language reduces the transaction costs of negotiating and interpreting agreements that bind end users to respect data use conditions. This reinforces existing efforts to align the data use conditions applied to data, through the adoption of common consent language and bioethics guidance, and the oversight applied to access requests, through the standardization of data access request forms and DAC processes.^31,32^ Such alignment has facilitated the development of software tools that enable the machine-representation and automated comparison of data use conditions, and the partial automation of DAC oversight. Complementary software tools will also be needed to enable the machine interpretation of contractual language and is the focus of our future work.^33,34^

## Supplementary Material

Supplementary Materials 1

Supplementary materials 2

The online version of this article (https://doi.org/10.1016/j.gim.2025.101594) contains supplemental material, which is available to authorized users.

## Figures and Tables

**Table 1:** EPS members provided substantive feedback and requested material changes to draft clauses during 4 virtual working group sessions

Changes Made in Collaboration With the EPS	Appendices or Clauses Affected
Incorporation of new appendices that further describe the data shared and detail the permitted uses thereof.	Data shared (Appendix 1 of the Model DAA). Data use conditions associated thereto (Appendix 1 of the Model DAA).
Introduction of additional boilerplate clauses to produce a full contractual agreement template.	Definitions. Identification of parties. Liability clause. Duration clause.
Clauses modified to incentivize further collaboration between data stewards and data users.	Contract breach notification. Data destruction. Data security. Data breach notification.
Clauses modified to incorporate additional clarifications on context-specific application, and to detail exceptions.	Intellectual property requirements. Reporting and monitoring requirements. Reidentification and harm. Data destruction.
Final list of clauses retained.	The categories of clauses included in the final review are as follows: (1) Definitions. (2) Purpose of use. (3) Reporting and monitoring of use and access. (4) Intellectual property requirements. (5) Outbound data transfers. (6) Contract breach notification. (7) Confidentiality. (8) Reidentification and harm. (9) Scientific publication. (10) Data destruction. (11) Data security. (12) Data breach notification. (13) Changes to the agreement. (14) Liability. (15) Duration and Termination.

*DAA*, data access agreement; *EPS*, Ethical Provenance Subgroup.

**Table 2:** Summary of DAA geographic provenance, and DAA population or disease area

Criterion	Number of Agreements
Geography	
Global	2
Oceania	2
North America	9
Europe (United Kingdom)	4
Europe (EU or EEA)	6
Asia	4
Africa	2
Population or disease area	
Administrative health data	1
Cancer	3
General	6
Genomic	13
Pediatric	1
Indigenous	2
Neurodegenerative disease	1
Neuroscience	2
Epidemiology	3
Personalized medicine	2

*DAA*, data access agreement.

## Data Availability

Record-level data that elaborate the core elements of each contract reviewed are available in the supplemental information. Multiple research institutions provided our team with copies of contracts and data access agreements on a strictly confidential basis. For this reason, we are unable to share the raw qualitative data sets used to inform the draft model clauses.
