# Supplementary material for: Toward ethical provenance tracking: The GA4GH model data access agreement (DAA)
Source: Genet Med. Author manuscript; Available in PMC 2026 Jun 19. (PMC13281905; doi:10.1016/j.gim.2025.101594)
Supplement: Supplementary Materials 1 [file NIHMS2180075-supplement-Supplementary_Materials_1.docx]

**Supplementary Information (1)**

**GA4GH MODEL DATA SHARING AGREEMENT (DSA)**

(1) [NAME INSTITUTION], (the “Data Provider”), a legal person duly incorporated pursuant to ..., having its head office at ... [name location],

AND:

(2) [NAME INSTITUTION], (“You” or “the User Institution”), a legal person duly incorporated pursuant to the laws of ____________________________________________, having its head office at

_________________________________________________________________________

AND:

Acknowledged by:

(3) [NAME PRINCIPAL INVESTIGATOR], (the “Principal Investigator”)

________________________________________ _________________________________________________________________________________

[Principal Investigator] (individually a “Party” and collectively the “Parties”)

1. **Definitions:**

**Agreement:** means the present agreement;

**Authorized Personnel**: means the employees or the agents responsible for performing the anticipated Research Project on behalf of the User Institution and the Principal Investigator;

**Authorized Trainees**: means the trainees that support the performance of the anticipated Research Project on behalf of the User Institution and the Principal Investigator;

**Data:** means the data, in whole or in part, that is made accessible to You by the Data Provider on the basis of this Agreement, including metadata, and any enriched or derived data which poses a serious risk of causing the re-identification of research participants within the context of its anticipated use. It includes, but is not limited to, representations, observations, narratives or measurements that could be used as the basis for further analysis, calculation or reasoning, regardless of the manner of their expression or the medium used to represent them;

**Data Provider:** means the legal entity, or organisation, that authorizes the User Institution, the Principal Investigator, and the Research Team access to access the Data, and acts as the formal data custodian responsible for performing oversight and stewardship functions relative to the Data;

**Principal Investigator:** means the senior researcher that has acknowledged this agreements and is accountable for ensuring the responsible conduct of the proposed Research Project, including the supervision of Authorized Personnel and Authorized Trainees;

**Research Team**: means the Principal Investigator, the Authorized Personnel, and the Authorized Trainees described in Appendix B of this Agreement;

**User Institution:** means the research organisation or legal entity that is made accountable for the responsible use of the Data, in accordance with the terms of this agreement, and all access approvals, policies, or supplementing materials that can reasonably be construed to condition the use thereof.

1. **Purposes of Use**

You agree to only use the Data shared with you for the approved purposes, in accordance with the conditions established in this Agreement. You further agree to respect restrictions on data use arising from public law that applies to You, research ethics guidance, contracts, informed consent materials, institutional policies, and data access committee approvals [include descriptions of data use limitations, copies of applicable policies, and other relevant supporting materials in Appendix A]. .

1. **Reporting and Monitoring of Use and Access**

You agree to submit to the Data Provider a report detailing the use made of the Data upon the completion of the approved Research Project, or upon termination of this Agreement, whichever comes first.

You agree to maintain a list detailing all of the members of the Research Team that will have access to the Data, including their name, their qualifications, and their role. You agree to notify the Data Provider of the addition or removal of Research Team members [The template in Appendix B can be used for this purpose]. .

You further agree that each member of the Research Team will be required to read and acknowledge this Agreement and to confirm their compliance with its conditions. The Principal Investigator is required to read and acknowledge the terms of the Agreement, to confirm their compliance with its conditions, and to monitor the compliance of the Research Team with its conditions.

1. **Intellectual Property Requirements**

You understand and acknowledge that the Data may be protected by copyright and other intellectual property rights. Duplication, as reasonably required to carry out Your Research Project with the Data, is nonetheless permitted. Sale of all or part of the Data on any media is not permitted.

You recognise that nothing in this Agreement shall operate to transfer to You any intellectual property rights in or relating to the Data.

You agree not to make intellectual property claims on these Data. You agree not to use intellectual property protection in ways that would prevent or block access to, or use of, any element of these Data, or conclusions drawn directly from these Data.

You can elect to perform further research that would add intellectual and resource capital to these Data and decide to obtain intellectual property rights on these downstream discoveries. In this case, You agree to implement licensing policies that will not obstruct further research and to respect the Fort Lauderdale Agreement and the Organisation for Economic Co-operation and Development Guidelines for the Licensing of Genetic Inventions [consider also listing other policies relevant to the local jurisdiction].

1. **Outbound Data Transfers**

You agree not to transfer or to disclose the Data to third parties that are not listed in Your Research Team. Should You wish to share the Data with an external collaborator outside of Your institution, the external collaborator must first obtain approval from the Data Provider to use the Data. The external collaborator must complete a separate Data Access Agreement authorizing them to use the Data.

Notwithstanding, the Data can be transferred or disclosed to third parties for the purpose of ensuring compliance with the monitoring and audit requirements that research funding agencies, sponsors, and regulators impose, or with binding public law. You may also disclose the data to third-party service providers that provide services that are integral to the performance of the research activities described in this agreement.

You agree to provide notice to the Data Provider of all transfers made to recipients other than third-party service providers at the time that these transfers are made.

In all cases, you must bind recipient third parties to hold the data according to standards of confidentiality and security that are equivalent to those described in this agreement.

1. **Contract Breach Notification**

If You are in breach of the terms of this Agreement, the Data Provider can terminate the Agreement at its discretion if: (i) the breach has not been remediated within a reasonable period of time after You become aware of such a breach, or (ii) the breach is serious and material, and cannot be remedied. A reasonable period of time shall generally be interpreted to mean thirty (30) days.

If You become aware that You are in breach of the terms of this Agreement, You must immediately notify the Data Provider.

1. **Confidentiality**

You agree to preserve, at all times, the confidentiality of the Data. You agree not to use or to attempt to use the Data in a manner that infringes the confidentiality of the Research Participants.

1. **Re-identification and Harm**

You agree not to attempt to re-identify Research Participants, nor to take any actions that could reasonably be expected to result in the re-identification thereof. You further agree not to attempt to contact or communicate with the Research Participants.

You agree not to use the data in a manner that is reasonably anticipated to cause Research Participants, their families, their communities, or members of specific populations to experience harm or stigmatization.

1. **Scientific Publication**

In publishing the findings from Your Research Project, You agree to include the attribution statement that is associated to the dataset, if any. Such an attribution statement provides clear instructions on how to attribute the Research Team, and/or the database or biobank of origin, and/or the dataset.

In addition, You agree that the publications arising from Your Research Project will not contain personal data of Research Participants. Such publications will not contain information that could reasonably be expected to cause a Research Participant to experience harm or stigmatization.

1. **Data Destruction**

You agree to destroy the Data once it is no longer useful for Your Research Project or upon termination of this Agreement, whichever comes first.

You agree not to retain any copies of the Data, except as required to ensure compliance with: applicable legal requirements; compliance with Data retention or audit requirements; or to preserve study integrity.

If you wish to proceed to the disposition of the Data through another means, such as its anonymisation, its archiving, or the return thereof to the Data Provider, you must obtain explicit approval from the Data Provider to do so.

1. **Data Security**

In handling the Data, You agree to implement and to maintain reasonable security measures that are appropriate to protect confidential and non-public information of a sensitive nature. Security measures must include the following: physical, organisational, and technological safeguards.

Upon written request, You agree to allow the Data Provider to audit the security and management documentation to ensure compliance with the terms of this Agreement.

1. **Data Breach Notification**

You agree to provide immediate notice to the Data Provider if you become aware of (or reasonably suspect) a data breach, unauthorised disclosure, or unauthorised use of the Data (together, breach).

You further agree to collaborate in good faith with the Data Provider to remedy and to mitigate the associated harms. You agree that You will stop processing the Data immediately upon the discovery of a data breach, until such a time as the You and the Data Provider agree to a course of remedial action and mutually decide to resume data processing.

You understand that the Data Provider may inform third parties such as regulatory authorities or affected individuals of the concerned breach, without being required to notify You.

1. **Changes to the Agreement**

You understand that the Data Provider may be required to alter the terms of this agreement to respond to changes in applicable legislation, to address evolving ethical and legal guidance, or to address other considerations that could emerge in the future. If this occurs, Your continued access to the Data, and use thereof, are conditional on the conclusion of a modified version of this agreement.

1. **Liability**

The Data is provided as-is, without warranties or guarantees, express or implied, as to its fitness for a particular purpose, its accuracy, quality, or comprehensiveness. The Data Provider accepts no liability or other responsibility for direct or indirect damages or losses arising from the use of the Data, nor for direct or indirect damages or losses occasioned due to the unavailability of the Data.

Each party agrees to bear all liability arising from its use, storage, and disposal of the Data. Each party shall be liable for any loss, claim, damage or liability that said party incurs as a result of its activities under this agreement. In all eventualities, no party to the agreement shall be required to provide compensation to the other that exceeds limits established in applicable public law.

1. **Duration**

This agreement has a duration of one year [if a different duration is intended, replace the foregoing text as needed]. The parties can agree to renew the agreement according to the same conditions in writing, prior to its conclusion. The agreement can be terminated earlier according to the conditions established in this agreement, or upon the mutual written consent of both parties.

**APPENDIX A: DESCRIPTION OF DATASETS AND DATA USE LIMITATIONS**

Please include a description of the data to which this agreement applies in this section, using the nomenclature that is preferred by your institution. This could include: digital object identifiers (DOIs), dataset identifiers from the data catalog of origin, or other.

Please include a description of the data use limitations applicable to the datasets shared. These can be included in the form of textual descriptions, or through reference to external policies or other supporting documents that provide descriptions of the applicable data use limitations. If no additional data use limitations other than those described in the contract are applicable to the shared data, this section need not be completed.

**APPENDIX B: RESEARCH TEAM MEMBERS**

**Principal Investigator:**

Name:

Title:

Position:

Institutional affiliation:

Institutional email address:

Contact email address:

Institutional Representative:

Name:

Title:

Position:

Institutional affiliation:

Institutional email address:

Contact email address:

**Authorised Personnel:**

List each according to:

Name:

Title:

Position:

Institutional affiliation:

Institutional email address:

Contact email address:

**Authorised Trainees:**

List each according to:

Name:

Title:

Position:

Institutional affiliation:

Institutional email address:

Contact email address:
